# A clinical research pathway towards developing new insights into cardiomyopathy

**DOI:** 10.1093/emph/eov024

**Published:** 2015-09-28

**Authors:** Daniel T. Blumstein, Janet Buckner, Sajan Shah, Shane Patel, Michael E. Alfaro, Barbara Natterson-Horowitz

**Affiliations:** ^1^Department of Ecology and Evolutionary Biology, University of California, 621 Charles E. Young Drive South, Los Angeles, CA 90095-1606, USA and; ^2^David Geffen School of Medicine at UCLA, Division of Cardiology, 650 Charles E. Young Drive South, A2-237, Los Angeles, CA 90095, USA

We were delighted to read Madias’ reply [1] to our comparative analysis [2], and we are excited that others see the value of making clinical predictions from a comparative life history study. We agree that further research on non-humans specifically aimed at identifying the mechanism(s) underlying capture myopathy would be valuable. The suggestions [1] to use echocardiography, electrocardiography, catecholamine and creatine phosphokinase measurements on non-humans would be very useful further identifying the precise links between capture myopathy in animals and takotsubo cardiomyopathy in humans. Furthermore, using life history traits in animal species most susceptible to capture myopathy to better understand individual vulnerability to takotsubo cardiomyopathy in humans is intriguing and worthy of further investigation.

Ultimately, we see great promise in comparative studies that help put human pathologies into a life history perspective and encourage others to consider how life history variation and trajectories may influence human health.
